# Structural Comparisons of Cefotaximase (CTX-M-ase) Sub Family 1

**DOI:** 10.3389/fmicb.2021.688509

**Published:** 2021-08-24

**Authors:** Ben A. Shurina, Richard C. Page

**Affiliations:** ^1^Department of Chemistry and Biochemistry, Miami University, Oxford, OH, United States; ^2^Cell, Molecular, and Structural Biology Program, Miami University, Oxford, OH, United States

**Keywords:** cefotaximase, serine-beta-lactamase, lactamase, CTX, diazabicyclooctane, boronic acid transition state analog inhibitor

## Abstract

The cefotaximase or CTX-M, family of serine-β-lactamases represents a significant clinical concern due to the ability for these enzymes to confer resistance to a broad array of β-lactam antibiotics an inhibitors. This behavior lends CTX-M-ases to be classified as extended spectrum β-lactamases (ESBL). Across the family of CTX-M-ases most closely related to CTX-M-1, the structures of CTX-M-15 with a library of different ligands have been solved and serve as the basis of comparison within this review. Herein we focus on the structural changes apparent in structures of CTX-M-15 in complex with diazabicyclooctane (DABCO) and boronic acid transition state analog inhibitors. Interactions between a positive surface patch near the active site and complementary functional groups of the bound inhibitor play key roles in the dictating the conformations of active site residues. The insights provided by analyzing structures of CTX-M-15 in complex with DABCO and boronic acid transition state analog inhibitors and analyzing existing structures of CTX-M-64 offer opportunities to move closer to making predictions as to how CTX-M-ases may interact with potential drug candidates, setting the stage for the further development of new antibiotics and β-lactamase inhibitors.

## Introduction

The use of β-lactam-based antibiotics has created substantial evolutionary pressure on bacteria. This pressure has resulted in the expression of a vast array of β-lactamases. Of these, extended spectrum β-lactamases (ESBL) have been a consistent point of concern for the clinical care of patients. One such family of ESBLs is the cefotaximase (CTX-M) family. The CTX-M-ases are classified as Ambler Class A (Ambler et al., 1991; Bush et al., 1995) or Jacoby-Bush Functional Group 2be (Bush and Jacoby, 2010). CTX-M-ases feature a catalytic serine at Ambler position 70 that hydrolyze β-lactam containing antibiotics after acylation. The initial discovery of members of the CTX-M family happened in Germany (Bauernfeind et al., 1990), France (Bernard et al., 1992), and Argentina (Bauernfeind et al., 1992). These newly identified β-lactamases featured amino acid sequences distinct from those of SHV and TEM, sharing less than 40% sequence identity. It was later discovered that CTX-M-1 was also identified, named, and characterized in parallel as MEN-1, as subsequent genetic sequencing revealed that these two enzymes were entirely identical (Barthélémy et al., 1992). The CTX-M-ase family was initially notable for its resistance to the third-generation cephalosporins, cefotaxime, and ceftazidime, while maintaining susceptibility to cefoxitin, latamoxef, or imipenem (Bernard et al., 1992). The initial members of the CTX-M-ase family were inhibited by β-lactamase inhibitors such as clavulanic acid (Bernard et al., 1992), though some newly identified variants have displayed resistance (Karim et al., 2001). In the first twelve years after the initial discovery of CTX-M-1, 36 CTX-M-ase variants were identified (Walther-Rasmussen and Høiby, 2004). At the time of writing, 246 members of the CTX-M family have been cataloged in the BLDB (Naas et al., 2017). The CTX-M family has been extraordinarily prolific and its global spread has been described by others (Bonnet, 2004; Naseer and Sundsfjord, 2011; Bevan et al., 2017).

## CTX-M-1 Family

The rapid identification of initial members of the CTX-M family, and enzymes of identical amino acid sequences described under differing naming conventions, predicated the need for phylogenetic classification, grouping, and renaming. These initial efforts produced several sub-families for the CTX-M-ases: CTX-M-1-like, CTX-M-2-like, CTX-M-8-like, CTX-M-9-like (Walther-Rasmussen and Høiby, 2004), and more recently CTX-M-25-like and KLUC-1-like (D’Andrea et al., 2013). The CTX-M-1-like family currently has 115 members. Of these, only CTX-M-15, CTX-M-64, and CTX-M-96/12a have publicly accessible structures deposited in the PDB. At time of writing, a total of 17 structures have been deposited for the CTX-M-1-like sub family of the CTX-M-ases (Naas et al., 2017).

While CTX-M-1 was the earliest identified member of the CTX-M-ases, no structure of CTX-M-1 has been deposited to the PDB. Of the CTX-M-1-like enzymes with deposited structures, CTX-M-15 is the closest in sequence identity (98.28%). Five mutations differentiate CTX-M-15 from CTX-M-1: V77A, D114N, S140A, D239G, and N286D. Of these five, V84A, D114N, and S140A are most broadly shared across the CTX-M-1-like sub-family (D’Andrea et al., 2013). CTX-M-64 then varies from CTX-M-15 by an additional 23 mutations. This review is focused on the structural features of CTX-M-1-like enzymes, thus due to the lack of a structure of CTX-M-1 itself and the corresponding wealth of structures of CTX-M-15, CTX-M-15 will largely serve as the basis for comparison for the enzymes in this review. When appropriate, differences and similarities to CTX-M-64 will be described.

## Structural Features of CTX-M-ases

**FIGURE 1:**
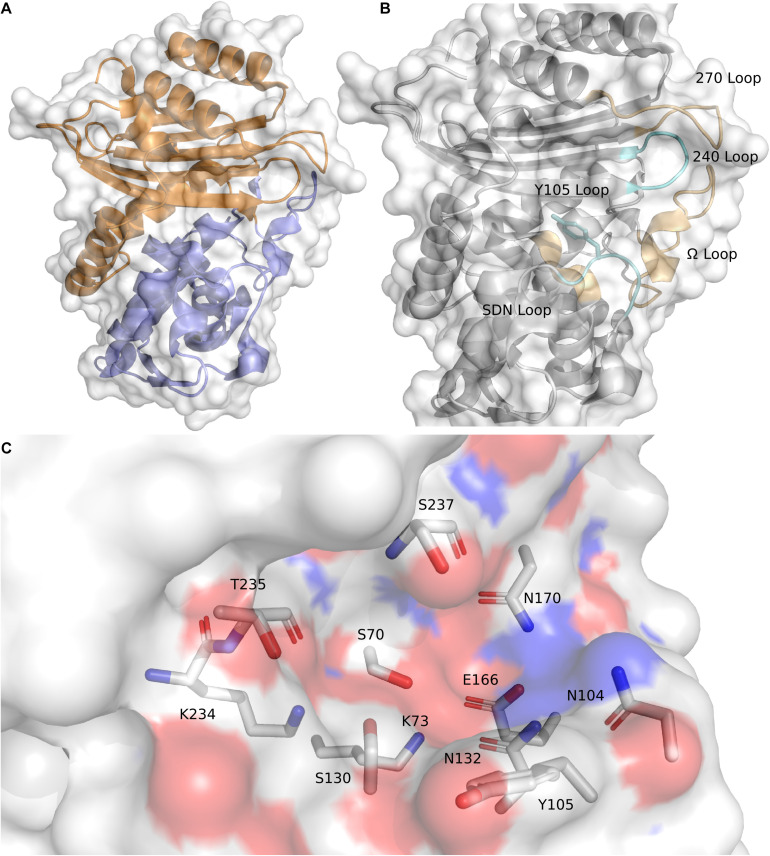
CTX-M-15 architecture. **(A)** The structure of CTX-M-15 (*white surface*) features two subdomains, an α + β sandwich domain (*orange*) and an α-helical domain (*blue*). **(B)** Similar to other Class A β-lactamases, CTX-M-15 features the conserved Ω-loop, the 240-loop, the 270-loop, and the SDN-loop. **(C)** The CTX-M-15 is lined with a set of canonical residues, include the active site serine S70.

The overall structure of CTX-M-15 is consistent with other Ambler Class A β-lactamases and the rest of the CTX-M-ase family (Ibuka et al., 2003; Lahiri et al., 2013). The protein features two sub-domains consisting of an α + β sandwich and an α-helical domain (Figure 1A). The α + β sandwich spans both the N- and C-terminal ends of the protein and harbors a core of five β-sheets. β-sheets 2 and 3 are separated in sequence space by the residues composing the helical cluster. Like other Class A β-lactamases, the conserved Ω-loop region is near the 240-loop, the 270-loop, and the SDN-loop (Jacob et al., 1990; Parker and Smith, 1993) (Figure 1B). The active site is formed by the surface cleft at the interface between the two sub-domains (Figure 1C). Residue C69 is conserved but is not involved in an active-site disulfide bridge as is observed in other Class A β-lactamases such as KPC-2 and SME-1 (Raquet et al., 1997; Majiduddin and Palzkill, 2003; Shurina and Page, 2019) and is in fact the only cysteine residue within the mature protein. The residue at the analogous position to C238 in CTX-M-15 is a glycine, and G238C mutants of CTX-M-15 appear unable to form disulfide bonds between C69 and C238 (Sabatini et al., 2017). At this stage it is also worth noting the structure of CTX-M-96 as the sequence of CTX-M-96 differs only from CTX-M-15 by two mutations, N89S and V276I, which are far from the active site. CTX-M-96 was originally described as a D240G mutant of CTX-M-12 (Mantilla Anaya et al., 2009), and the deposited crystal structure and extensive enzymatic characterization of CTX-M-96 show similar structure and behavior to CTX-M-15 (Ghiglione et al., 2015). Alignment of the apo structure of CTX-M-15 with the structure of CTX-M-96 produces a backbone RMSD of 0.159 Å indicating high structural similarity.

Consistent with other Class A β-lactamases, such as SHV and TEM, residue 105 is a tyrosine. The residue at position 105 typically features a conjugated ring system that is thought to play a role in substrate recognition, possibly through π–π stacking (Doucet and Pelletier, 2007; Li et al., 2012; Singh and Dominy, 2012; Klein et al., 2018). The sidechain of N104 in the apo structure of CTX-M-15 is located spatially near the sidechain of N132, a residue within the SDN-loop. These two residues comprise a portion of the cationic patch in the active site that interacts with carboxamide carbonyl oxygens attached to the core β-lactam architecture (Lahiri et al., 2013). Disruption of this cationic patch by mutating N132 appear to significantly impair substrate binding and catalytic function (Ourghanlian et al., 2017). The core catalytic residues are S70, to which substrate is acylated, and the three main residues involved in the proton shuttling mechanism implicated in β-lactam hydrolysis: S130, E166 (itself part of the Ω-loop), and K73 (Chen et al., 2005, 2007). The sidechains of these residues work in conjunction with tightly coordinated networks of water molecules. The backbone of S70, in conjunction with the backbone atoms of S237, forms the “oxyanion hole” (Murphy and Pratt, 1988). S237 and G240 also appear to be key for the activity profile of the cefotaximase family (Chen et al., 2007; Delmas et al., 2008). Immediately preceding S237 are the residues that constitute the conserved KTG motif (Joris et al., 1991). The KTG motif and the S70XXK73 motif are found in both penicillin binding proteins and serine β-lactamases.

Of the 10 published CTX-M-15 structures (Table 1), six contain a diazabicyclooctane (DABCO) based inhibitor (Lahiri et al., 2013; King et al., 2015, 2016; Tooke et al., 2019b), and three contain boronic acid transition-state analog inhibitors (BATSIs) (Hecker et al., 2015; Cahill et al., 2017; Liu et al., 2020). None of the current structures of CTX-M-15, or any members of the CTX-M-1-like family contain structures with natural substrates or their subsequent acyl intermediates, unlike those deposited for members of other CTX-M families (Shimamura et al., 2002; Chen et al., 2005), including CTX-M-64. Later portions of this review focus, in part, on features of published CTX-M-15 structures featuring DABCOs or boronic acid transition state analog inhibitors, and structures of acyl-intermediates of CTX-M-64 in complex β-lactam inhibitors (Table 1).

**TABLE 1:** Summary of CTX-M subfamily 1 structures.

**Enzyme**	**PDB ID**	**Year released**	**Resolution**	**Structure type**	**Active site ligand**	**Potency or binding data**
CTX-M-15	4HBT	2013	1.1	apo	None	NA
CTX-M-15	4HBU	2013	1.1	Diazabicyclooctane	Avibactam	*K*_*d*_ 0.002 uM (Ehmann et al., 2013)
CTX-M-15	4S2I	2015	1.6	Diazabicyclooctane	Avibactam	*K*_*d*_ 0.002 uM (Ehmann et al., 2013)
CTX-M-15	4XUZ	2015	1.5	Boronic acid transition-state analog	RPX-7009 (vaborbactam)	*K*_*i*_ 0.044 uM (Hecker et al., 2015)
CTX-M-15	5FA7	2016	1.67	Diazabicyclooctane	FPI-1523	*K*_*d*_ 0.004 uM (King et al., 2016)
CTX-M-15	5FAO	2016	3.01	Diazabicyclooctane	FPI-1465	*K*_*d*_ 0.011 uM (King et al., 2016)
CTX-M-15	5FAP	2016	2.7	Diazabicyclooctane	FPI-1602	*K*_*d*_ 0.010 uM (King et al., 2016)
CTX-M-15	5T66	2017	1.95	Boronic acid transition-state analog	1C	IC_50_ 1.7 ± 0.1 (nM) (Cahill et al., 2017)
CTX-M-15	6QW8	2019	1.1	Diazabicyclooctane	Relebactam	IC_50_ 400 (nM) (Tooke et al., 2019b)
CTX-M-15	6SP6	2020	1.1	Boronic acid transition-state analog	Taniborbactam (VNRX-5133)	IC_50_ 0.01 uM (Liu et al., 2020)
CTX-M-64	5ZB7	2019	1.63	apo	None	N.A.
CTX-M-64	6ITY	2019	2.14	β-lactam containing	Sulbactam	IC_50_ 0.365 nM (Cheng Q. et al., 2019)
CTX-M-64 (TDN)	6J25	2019	1.2	apo-Mutant	None	NA
CTX-M-64 (TDN)	6J2B	2019	1.44	β-lactam containing-mutant	Sulbactam	IC_50_ 2.21 × 10^6^ nM (Cheng Q. et al., 2019)
CTX-M-64 (TDN)	6J2K	2019	1.44	β-lactam containing-mutant	Clavulanic acid	IC_50_ 4.29 × 10^1^ nM (Cheng Q. et al., 2019)
CTX-M-64	6J2O	2019	1.9	β-lactam containing	Clavulanic acid	IC_50_ 2.28 nM (Cheng Q. et al., 2019)
CTX-M-96	3ZNY	2014	1.2	apo	None	NA

## Emergence and Similarity of CTX-M-15 and CTX-M-64

CTX-M-15 was first described in 2000 from clinical isolates from patients in India as a derivative of CTX-M-3 (Karim et al., 2001). Shortly thereafter CTX-M-15 was again identified in clinical isolates from patients in Poland (Baraniak et al., 2002). CTX-M-15 rapidly became one of the predominant variants of the CTX-M-1-like sub-family in clinical (Muzaheed et al., 2008; Nicolas-Chanoine et al., 2008; Peirano et al., 2010; Lee et al., 2011), agricultural (Haenni et al., 2016; Diab et al., 2017), and natural (Mohsin et al., 2017) settings. CTX-M-64 was first identified in *Shigella sonnei* clinical isolates from patients in Japan in 2006 (Nagano et al., 2009). Initial sequencing of CTX-M-64 suggested that the protein was a recombinant fusion construct of CTX-M-15 and CTX-M-14 (Nagano et al., 2009). CTX-M-14 itself has been identified in parallel under the names CTX-M-14 (Pai et al., 2001), CTX-M-18 (Poirel et al., 2001; Cantón and Coque, 2006), KLUY-1 (Olson et al., 2005), TOHO-3, and UOE-2 (Ishii et al., 2005). CTX-M-14, part of the CTX-M-9-like family, may be an evolutionary progenitor of the sub-family based on its prevalence and genetic composition. When CTX-M-64 was discovered, CTX-M-14 and CTX-M-15 were the two most prevalent CTX-Mases in the clinic (Cantón et al., 2012). Co-expression of a variety of CTX-M enzymes is not uncommon in patients, and horizontal gene transfer between bacterial vectors in different host organisms has been implicated as a partial source for this phenomena (Sun et al., 2010).

**FIGURE 2:**
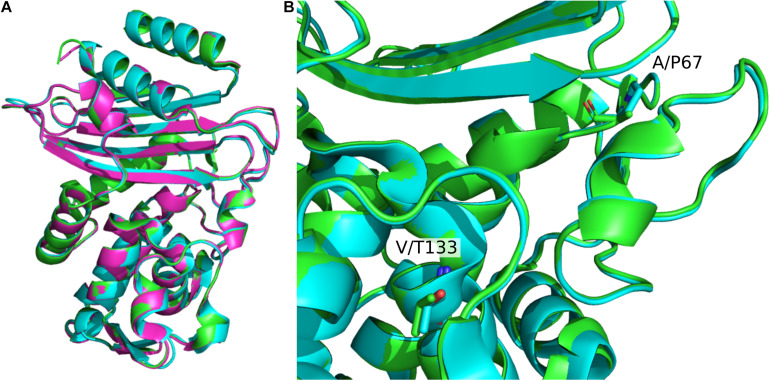
Structural alignment of CTX-M-1-sub family apo structures. **(A)** Alignment of CTX-M-15 (green, 4HBT) CTX-M-64 (blue, 5ZB7), and CTX-M-96 (pink, 3ZNY). Global β-lactamase fold architecture is conserved. **(B)** Comparison of CTX-M-15 and CTX-M-64 A67P and V133T mutations. These two mutations are the closest to the active site. Other mutations are mainly located in the α-helical bundle.

**FIGURE 3:**
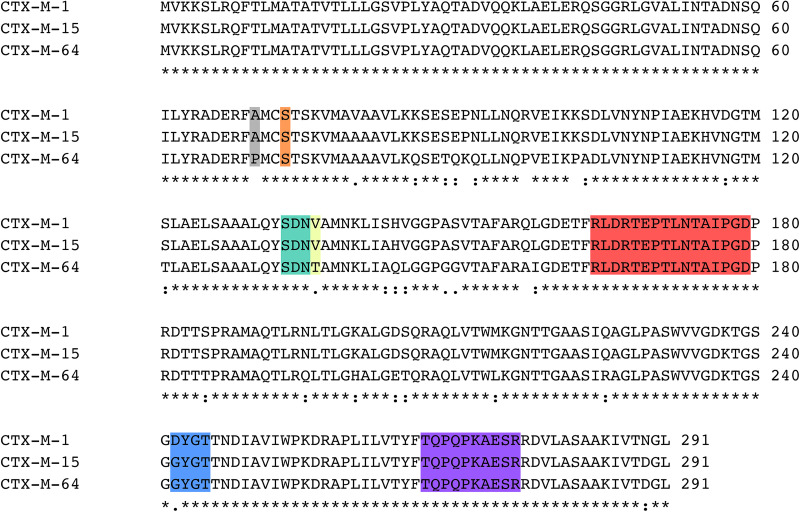
Sequence alignment of CTX-M-1, CTX-M-15, and CTX-M-64. Key regions of the sequence are highlighted, including the A67P mutation site (*gray*), Ser70 (*orange*), the SDN-loop (*green*), the V133T mutation site (*yellow*), the Ω-loop (*red*), the 240-loop (*blue*), and the 270-loop (*purple*).

A structural alignment of the apo structures of CTX-M-15 (4HBT) and CTX-M-64 (5ZB7) reveals a backbone RMSD of 0.286 Å, with only minor structural differences found (Figure 2A). The α-helical bundle region of CTX-M-64 is most similar to CTX-M-14. Analysis of the sequence alignment of CTX-M-64 compared to CTX-M-15, and CTX-M-1 reveals 91% identity (Figure 3). The N- and C-terminal ends of CTX-M-64 are most CTX-M-15-like, with most of the active site architecture, the Ω-loop, and β-sheet assembly being conserved. Like CTX-M-15, CTX-M-64 is capable of conferring resistance to ceftazidime (Nagano et al., 2009). Two mutations, A67P and V133T (Figure 3), are somewhat close to the active site but are not active participants (Figure 2B).

## CTX-M-15 DABCO Structures

Diazabicyclooctane derivatives are non β-lactam inhibitors of β-lactamases that have been undergoing continuous development since the mid-1990s (Coleman, 2011). When co-administered with mainline β-lactam antibiotics, DABCO inhibitors have proven to be effective against bacteria expressing Class A (Bonnefoy et al., 2004; King et al., 2015), C (Jacoby, 2009), and D (King et al., 2015) β-lactamases. New developments with DABCO compounds have produced DABCO based drugs that are capable of both the inhibition of β-lactamases, and penicillin binding proteins (King et al., 2016; Laws et al., 2019; Tooke et al., 2019a).

**FIGURE 4:**
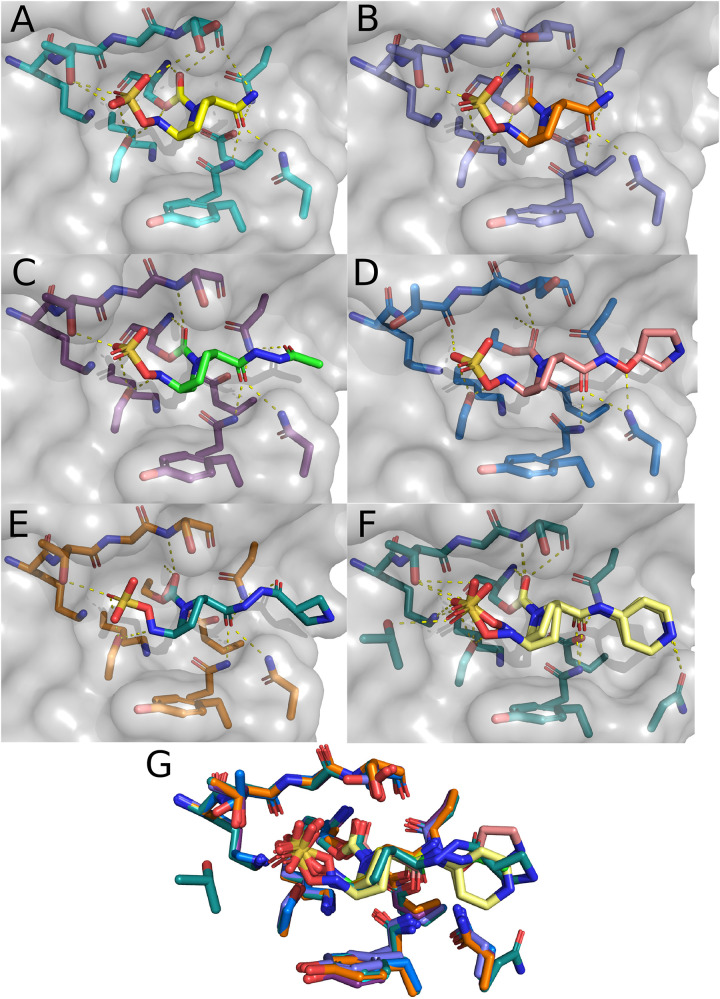
CTX-M-15 structures in complex with DABCOs. **(A)** Structure of CTX-M-15 with bound Avibactam (4HBU). **(B)** Alternate structure of CTX-M-15 bound to Avibactam (4S2I). **(C)** Structure of CTX-M-15 and FPI-1523 (5FA7). **(D)** Structure of CTX-M-15 in complex with FPI-1465 (5FAO). **(E)** Structure of CTX-M-15 and FPI-1602 (5FAP). **(F)** Structure of CTX-M-15 in complex with Relebactam. **(G)** Overlay of active site residues of CTX-M-15 and all DABCO structures.

**FIGURE 5:**
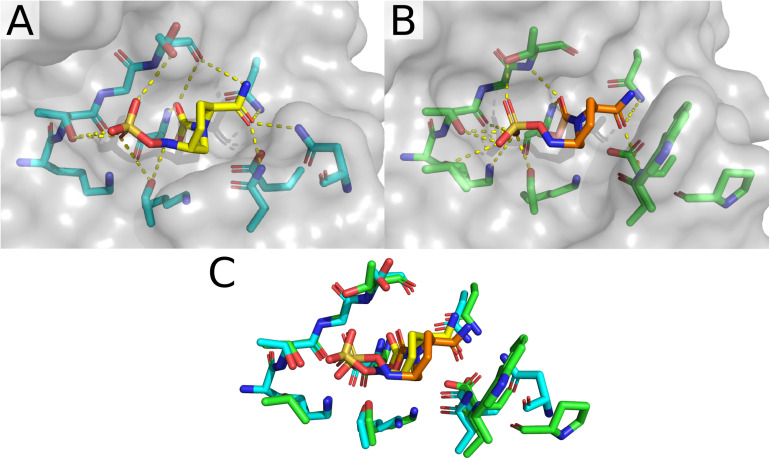
Avibactam bound to CTX-M-15 (4HBU) and KPC-2 (4BZE). **(A)** Structure and active site organization of avibactam-bound CTX-M-15. **(B)** Structure and organization of the active site of KPC-2. **(C)** Overlay of the active sites for avibactam-bound CTX-M-15 and KPC-2.

Comparing the two structures of CTX-M-15 complexed with avibactam (Figures 4A,B), there are only minute differences between the two in the side chain and Cα positions of residues involved in the active site. Changes in the structure of active site residues relative to the prior apo structure are also minimal. In 4S2I, S237 is depicted as only engaged in a single conformation where the side chain is engaged in polar contacts with the sulfate group on avibactam as well as the newly formed carboxyl group being generated from the acylation of avibactam (Figure 4B). In contrast, in 4HBU the side chain of S237 is engaged in polar contact with only the sulfate group and only with 50% occupancy (Figure 4A). The alternate conformation for S237 in 4HBU is rotated toward the Ω-loop and away from the substrate. Looking at the composite image of these two structures, the sidechain of S237 appears to be able to freely rotate to engage or disengage with substrates. The sidechain positions of Y105 are almost identical, and while they do not make direct contact with the acylated avibactam, they are involved in water-bridged contacts between the phenoxide of Y105 and the sulfate of avibactam. The carboxamide group in avibactam is held in place by the side chains of N104, N132 of the SDN-loop, and N170 of the Ω-loop. This partially positive surface comprised of the side chains of N104, N132, and N170 is where most of the structural differences in the active site across the set of CTX-M-15 structures are found. The presence of N104 in CTX-M-15 allows for coordinated interactions with the R groups connected to the carboxamide (Figure 5). This binding modality is not available to other Ambler Class A β-lactamases such as KPC-2 which has a proline at position 104. The lack of a possible route for forming polar contacts with residue 104 in KPC-2, and the positioning of the backbone atoms of residue 237 (Figure 5B), provides less stable interactions with the carboxamide and it’s substituents, which may explain some of the observed differences in the binding characteristics of DABCO drugs in members of the KPC family and that of CTX-M (Ehmann et al., 2013) (Figure 5).

Interactions between the carboxamide, its substituents, and this positive surface patch in CTX-M-15 are also seen in the structures containing the FPI-1465 (Figure 4D), FPI-1523 (Figure 4C), and FPI-1602 (Figure 4E) compounds. In all three structures N104 is engaged in polar contacts with the carboxamide. In the FPI-1465 structure, N104 makes a second polar contact with the oxy-group in the R substituent of the carboxamide, seemingly reorienting the carboxamide itself away from N170 and preventing the formation of potential polar contacts. In FPI-1523 and FPI-1602 these interactions with the main carboxamide are restored and accompanied by a secondary interaction with a carboxamide found within the R group. Polar contacts with N170 may act as an additional impediment to mobility of the Ω-loop, which if such motions are required for these enzymes to undergo acylation and deacylation of product and substrate, may partially explain the apparent differences in *K*_*d*_ for these compounds. The last remaining CTX-M-15 DABCO structure is with relebactam where the N104 side chain is rotated away from the carboxamide itself and is instead engaged in polar contacts with the nitrogen in the piperidine of the R group (Figures 4F,G).

## CTX-M-15 Boronic Acid Transition State Analogs

**FIGURE 6:**
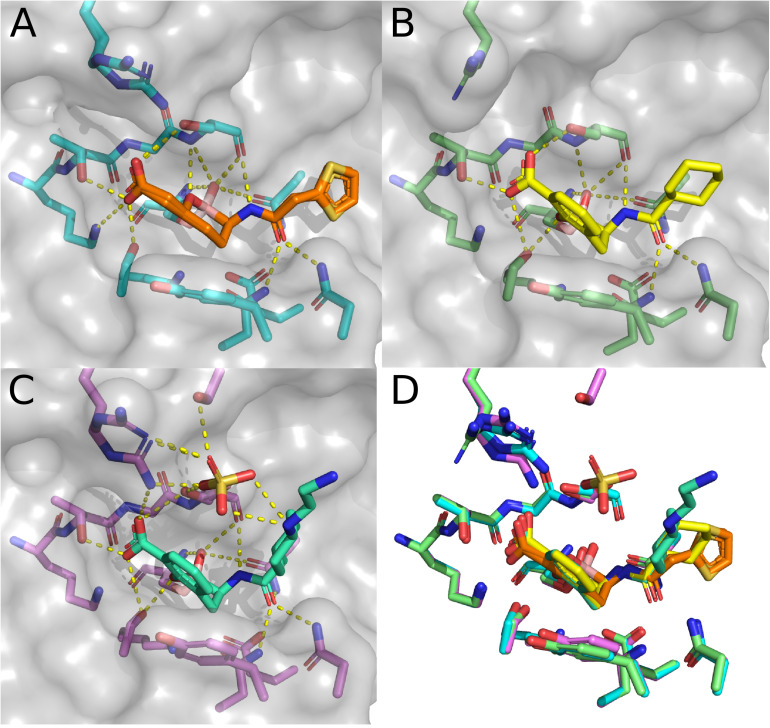
Structures in complex with BATSIs. **(A)** Structure of CTX-M-15 in complex with vaborbactam (4XUZ). **(B)** Structure of CTX-M-15 in complex with 1C (5T66). **(C)** Structure of CTX-M-15 in complex with taniborbactam (6SP6). **(D)** Overlay of the active sites for CTX-M-15 in complex with vaborbactam, 1C, and taniborbactam.

The final sub-set of CTX-M-15 structures are those containing BATSIs. Boronic acid derivatives have been explored as β-lactamase inhibitors for over 30 years (Bone et al., 1987). Modeled off of initial developments for the inhibition of serine-proteases (Lindquist and Terry, 1974), BATSI have effectively inhibited a variety of Ambler Class A β-lactamases (Beesley et al., 1983; Crompton et al., 1988; Caselli et al., 2001). Some of the first structures of BATSIs bound to Class A β-lactamases were of TEM-1 (Strynadka et al., 1996), SHV (Thomson et al., 2007), and KPC-2 (Ke et al., 2012). Beyond the strategy of BATSI-based drugs designed for the inhibition of serine β-lactamases, boron centers have been incorporated to overcome antimicrobial resistance through several other strategies nicely reviewed elsewhere (Krajnc et al., 2019). Prior to structural depositions of BATSIs bound to members of the CTX-M-1-like subfamily, several structures of BATSIs bound to CTX-M-2 (Tomanicek et al., 2013) and CTX-M-9 (Chen et al., 2005) were deposited. The first structure of CTX-M-15 bound to a BATSI was solved in 2015 at 1.5 Å resolution (Hecker et al., 2015) for the complex with RPX7009, now named vaborbactam (Figure 6A). The general binding modality of vaborbactam in the CTX-M-15 structure follows typical trends seen in structures of Class A β-lactamases. The tetrahedral boronic acid derivative replaces the carbonyl carbon found in the β-lactams found in natural substrates, with two of the oxygens connected to the boron center participating in polar contacts with the oxyanion hole. One of these oxygens also is close enough to interact with the sidechain of N170. In place of the sulfonates found in the CTX-M-15 DABCO structures, vaborbactam has a carboxylic acid that makes polar contacts with the side chains of S130, K234, T235, and S237. Like in the DABCO structures, N104, N132, and N170 are engaged in polar contacts with a carboxamide, but the relative placement of the carbonyl and amino groups are reversed. This reversal allows for polar contacts between the amino group and the backbone atoms of S237. Two conformers have been modeled in for this structure, which are primarily differentiated by the relative positioning of the thiophene group. Neither thiophene conformation provides direct contact with CTX-M-15, likely contributing to the multiple observed conformations within the crystal structure.

The second CTX-M-15 structure with a BATSI bound in the active site is for the compound referred to as “1C.” Essentially, the binding modality of 1C is quite similar to that of vaborbactam, with a possible exception of the presence of π–π stacking interactions between Y105 and the benzoxaborinine group found in 1C (Figure 6B). The third and final BATSI-CTX-M-15 structure contains taniborbactam (Figures 6C,D). This structure is the highest resolution structure of the three BATSI structures at 1.1 Å, and exhibits a few differences compared to those with 1C or vaborbactam. In the taniborbactam structure, an additional sulfate ion mediates interactions between CTX-M-15 and taniborbactam. While sulfates have been found in the active sites of crystal structures in CTX-M-15, as well as in peripheral surface contacts on the protein, this structure is the first amongst the CTX-M-1-like subfamily where these sulfate molecules interact with the substrate. The sulfate in this structure interacts with a secondary amine in taniborbactam and makes contacts with the side chains of S237, S272, and R274. The interactions with R274 bring the sidechain close enough to interact with the taniborbactam carboxylic acid. R274 appears to sample a variety of conformations across CTX-M-15 structures and occasionally makes contacts with the side chains of S237 and S272. Whether the contacts R274 make in the taniborbactam structure are biologically relevant, or a product of the crystallographic conditions containing 2.4 M ammonium sulfate is a matter that has not been fully investigated, nor has the role of R274 itself been much explored. R274 is, interestingly, conserved throughout most of the CTX-M-1-like subfamily. Only CTX-M-88 has a substitution at this position, replacing arginine with histidine (Ranjbar et al., 2010).

## CTX-M-64 Structures With β-Lactam Inhibitors

**FIGURE 7:**
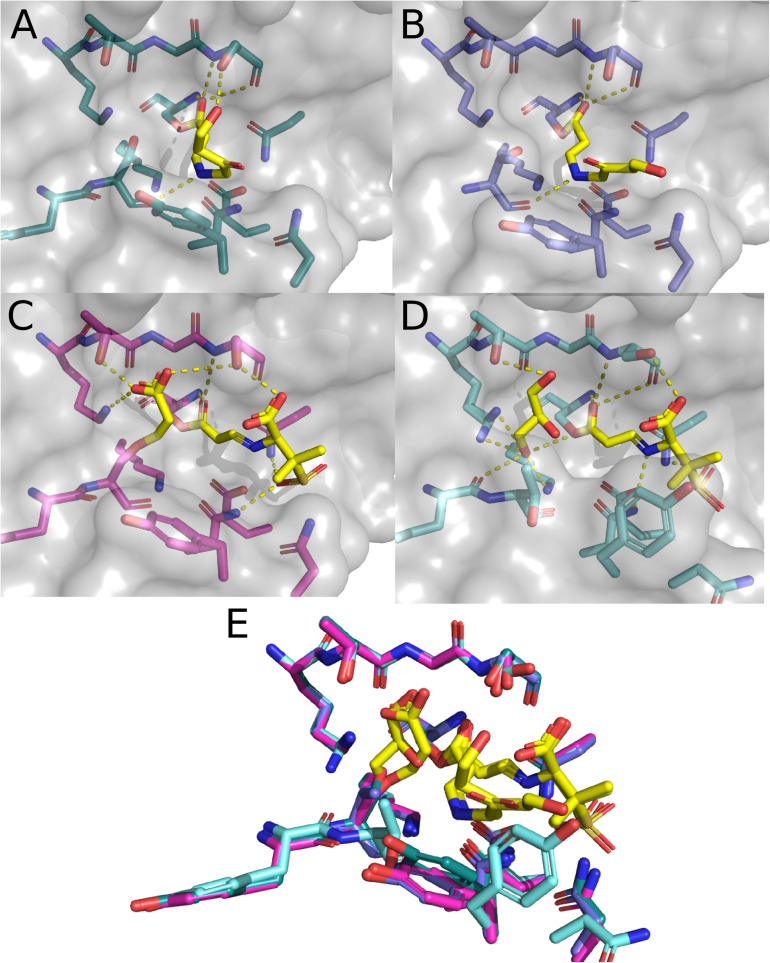
Structure of CTX-M-15 with Sulbactam and clavulanic acid. **(A)** CTX-M-64 complexed with hydrolysis products of clavulanic acid (6J2O). **(B)** CTX-M-64 TDN mutant bound to clavulanic acid hydrolysis products (6J2K). **(C)** CTX-M-64 bound to sulbactam hydrolysis products (6ITY). **(D)** CTX-M-64 TDN mutant bound to sulbactam hydrolysis products (6J2B). **(E)** Overlay of the CTX-M-64 and TDN mutant structures featured in panels **(A–D)**.

The enzymatic characterization of CTX-M-64 suggests that CTX-M-64 behaves in a similar manner to CTX-M-15. Building upon the apo structure of CTX-M-64 (5ZB7), two additional structures of CTX-M-64 have been deposited in the PDB. These structures contain sulbactam and clavulanic acid hydrolysis products. These are currently the only structures of members of the CTX-M-1-subfamily bound to any sort of natural substrate as sulbactam and clavulanic acid are β-lactam containing inhibitors (Weber et al., 1984; Campoli-Richards and Brogden, 1987). In the original work describing these structures, a clear mechanistic description of how these products are formed is provided and is supported by mass spectrometry data (Cheng Q. et al., 2019). The structure of a trans-enamine derivative of acylated clavulanic acid (Figure 7A) exhibits polar contacts made with the backbone and side chain of S237, as well as the backbone carbonyl of S130. The trans-enamine could then be hydrolyzed, and cross-linked to the side chain of S130. Evidence of this cross-linking pathway is also provided through the structure of CTX-M-64 with sulbactam hydrolysis products (Figure 7C). In this structure, a trans-enamine derivative of sulbactam is acylated to S70, making polar contacts with the oxyanion hole as well as the side chains of N132, N170, and S237. An acrylic acid is also acylated to the side chain of S130, which makes additional polar contacts with the side chains of K234, T235, and S237. The apo, sulbactam hydrolysis product, and clavulanic acid hydrolysis product structures were directly compared to CTX-M-64 S130T mutants. These mutants were described as “CTX-M-199-like” (Cheng Q. et al., 2019) as only two mutations differentiated CTX-M-64 from CTX-M-199: A109T and S130T (Cai et al., 2017). CTX-M-199 and the CTX-M-199-like mutants show an increased resistance to sulbactam and tazobactam (Cai et al., 2017; Cheng Q. et al., 2019).

The structure of clavulanic acid hydrolysis intermediates bound to CTX-M-64 S130T reveals a similar set of conformations relative to the CTX-M-64 structure (Figure 7B). The polar contacts between this intermediate and the protein are much the same, but the conformation of the intermediate itself is different. The CTX-M-64 S130T sulbactam derivative structure presents a different story (Figure 7D). Here, nothing is acylated to the hydroxyl in the side chain of residue 130. Instead, the hydroxyl is rotated away from the active site, and the backbone atoms are rotated such that the carbonyl points further into the active site cleft. This small shift results in two considerable changes. First, the rotation of the backbone carbonyl of T130 allows for concerted coordination of a glycerol moiety found in the active site. This glycerol makes additional contacts with the backbone atoms of Y129, as well as the side chains of K73, K234, and T235. The glycerol is likely an artifact of the crystallization conditions or cryoprotectant solution. The second and perhaps more important outcome from the shifts in T130 backbone atoms are the rearrangements of the rest of the “TDN”-loop as well as the Y105-loop. These additional shifts of the N132, N104, and Y105 residues push them out of the optimal range and geometry to make polar contacts with the sulbactam a trans-enamine derivative. The resulting reduction in direct interactions with the protein has been proposed as a mechanism by which the CTX-M-64 S130T, and by extension CTX-M-199 may confer resistance to inhibitors like sulbactam and tazobactam.

## Conclusion and Outlook

With 115 members in the CTX-M-1-like subfamily and structures of only four variants, there is obvious room for expansion in this niche. Much information can and has been inferred through comparisons with structures of CTX-M-2-like and CTX-M-9-like members of the greater CTX-M family, but the sheer number of CTX-M variants has left giant gaps throughout the entire domain. Within the set of structures deposited for the CTX-M-1-like subfamily, there are useful structural and mechanistic insights as to what role residues in the Ω-loop, the 240-loop, the Y105-loop, and the SDN-loop play in substrate and inhibitor binding. We are even able to garner glimpses of the dynamic landscape available to these enzymes. Further characterization of the dynamics of these enzymes could be accomplished via crystallization by using E166 mutants as was done for other Ambler Class A β-lactamases (Tooke et al., 2021). Extension into other techniques ideally suited for the characterization of dynamic processes like NMR and EPR would likely provide further insight, and such approaches have already proven successful for other serine β-lactamases (Savard and Gagné, 2006; Morin and Gagné, 2009; Griffin et al., 2011; Staude et al., 2016). Additionally, a whole-family approach to characterization, as has been done for B1 metallo-β-lactamases like NDM (Stewart et al., 2017), VIM (Cheng Z. et al., 2019; Thomas et al., 2020), and IMP (Salimraj et al., 2019; Cheng et al., 2021), may be appropriate. The prevalence and sheer number of CTX-M enzymes requires some sort of broad-scope investigation of the behavior, stability, and evolutionary pressures in this space. Knowing how and why these enzymes behave the way they do and gaining some predictive capacity as to what these enzymes might do in the future will be essential for the further development of new antibiotics and β-lactamase inhibitors.

## Author Contributions

BAS and RCP conceived of the project and wrote the manuscript. Both authors contributed to the article and approved the submitted version.

## Conflict of Interest

The authors declare that the research was conducted in the absence of any commercial or financial relationships that could be construed as a potential conflict of interest.

## Publisher’s Note

All claims expressed in this article are solely those of the authors and do not necessarily represent those of their affiliated organizations, or those of the publisher, the editors and the reviewers. Any product that may be evaluated in this article, or claim that may be made by its manufacturer, is not guaranteed or endorsed by the publisher.
